# Supplementary material for: Unpacking the Complexity of COVID-19 Fatalities: Adverse Events as Contributing Factors—A Single-Center, Retrospective Analysis of the First Two Years of the Pandemic
Source: Viruses. 2023 Jun 24;15(7):1430. doi: 10.3390/v15071430 (PMC10383259; doi:10.3390/v15071430)
Supplement: Supplementary file 1 [file viruses-15-01430-s001.zip › viruses-2462201-supplementary.pdf]

Table S1. Characteristics of selected parameters in different age groups

variable	<50 (N=21)	51-60 (N=46)	61-70 (N=131)	71-80 (N=128)	≥81 (N=151)	p-value
**Sex**						0.093
Women	5 (23.8%)	17 (37.0%)	49 (37.4%)	49 (38.3%)	74 (49.0%)	
Men	16 (76.2%)	29 (63.0%)	82 (62.6%)	79 (61.7%)	77 (51.0%)	
**Ward of admission**						**<0.001**
IDU	18 (85.7%)	36 (78.3%)	108 (82.4%)	114 (89.1%)	145 (96.0%)	
ICU	3 (14.3%)	10 (21.7%)	23 (17.6%)	14 (10.9%)	6 (4.0%)	
**Ward where the patient died**						**<0.001**
IDU	5 (23.8%)	10 (21.7%)	20 (15.3%)	51(39.8%)	127 (84.1%)	
ICU	16 (76.2%)	36 (78.3%)	111 (84.7%)	77 (60.2%)	24 (15.9%)	
Duration of symptoms before admission (days)	7.74 (4.83), 7 [4-10]	7.41 (3.71), 7 [5-9]	7.82 (4.75), 7 [5-10]	7.18 (5.24), 7 [4-8]	6.8 (4.07), 7 [5-7.5]	0.3
Length of hospitalisation (days)	18.52 (10.83), 19 [9-25]	16.15 (12.42), 13.5 [7.25-20.75]	16.53 (11.25), 16 [8.5-23]	14.45 (11.82), 12 [8-19.25]	9.72 (7.52), 8 [4-14]	**<0.001**
Comorbidities	18 (85.7%)	40 (87.0%)	115 (87.8%)	119 (93.0%)	144 (95.4%)	0.075
Coinfections	15 (71.4%)	30 (65.2%)	99 (75.6%)	76 (59.4%)	54 (35.8%)	**<0.001**
**COVID-19 complications**						
Pulmonary embolism	1 (4.8%)	2 (4.3%)	11 (8.4%)	13 (10.2%)	19 (12.6%)	0.5
Ischaemic stroke	0 (0%)	4 (8.7%)	6 (4.6%)	8 (6.2%)	10 (6.6%)	0.7
Myocardial infarction	1 (4.8%)	3 (6.5%)	5 (3.8%)	8 (6.2%)	5 (3.3%)	0.7
Limb ischaemia	3 (14.3%)	2 (4.3%)	8 (6.1%)	1 (0.8%)	2 (1.3%)	**0.004**
Bleeding complications	5 (23.8%)	13 (28.3%)	23 (17.6%)	16 (12.5%)	14 (9.3%)	**0.014**
Acute kidney injury	5 (23.8%)	13 (28.3%)	33 (25.2%)	38 (29.7%)	60 (39.7%)	0.092
Exacerbation of chronic heart disease	5 (23.8%)	10 (21.7%)	38 (29.0%)	26 (20.3%)	21 (13.9%)	**0.035**
Decompensation of liver function	5 (23.8%)	4 (8.7%)	1 (0.8%)	1 (0.8%)	1 (0.7%)	**<0.001**
**Complications of hospitalisation**						
Hospital-acquired infection	12 (57.1%)	21 (45.7%)	55 (42.0%)	47 (36.7%)	24 (15.9%)	**<0.001**

Emphysema and other complications of ventilation	2 (9.5%)	6 (13.0%)	16 (12.2%)	10 (7.8%)	2 (1.3%)	**0.001**
Trauma, fall	2 (9.5%)	1 (2.2%)	5 (3.8%)	5 (3.9%)	12 (7.9%)	0.3
Predominant cause of death – COVID-19 respiratory failure	17 (81.0%)	34 (73.9%)	119 (90.8%)	114 (89.1%)	139 (92.1%)	**0.012**
**Other causes of death**						
Malignant neoplasm	3 (14.3%)	1 (2.2%)	4 (3.1%)	4 (3.1%)	3 (2.0%)	0.13
Sudden cardiac arrest	4 (19.0%)	10 (21.7%)	16 (12.2%)	13 (10.2%)	13 (8.6%)	0.13
Surgical complications	1 (4.8%)	2 (4.3%)	0 (0%)	3 (2.3%)	1 (0.7%)	**0.047**
Lack of specialist treatment	2 (9.5%)	5 (10.9%)	7 (5.3%)	21 (16.4%)	29 (19.2%)	**0.006**
Septic shock	2 (9.5%)	8 (17.4%)	15 (11.5%)	8 (6.2%)	8 (5.3%)	0.054
Other	3 (14.3%)	2 (4.3%)	4 (3.1%)	2 (1.6%)	6 (4.0%)	0.1

Patients in the age group <50 years (N=21) had the following conditions: 9 patients with obesity, 2 with chronic kidney disease including one with Goodpasture syndrome, 4 with malignancy (hepatocellular carcinoma, acute myeloid leukaemia, breast cancer), 4 with arterial hypertension, 4 with type two diabetes, 2 with asthma, 1 with alcoholic cirrhosis.
